# Women’s experiences of childbirth during the COVID-19 pandemic in Lithuania

**DOI:** 10.18332/ejm/185750

**Published:** 2024-04-24

**Authors:** Alina Liepinaitienė, Gabija Čirbaitė, Julijana Stepanova, Vaidas Jotautis, Audrius Dėdelė

**Affiliations:** 1 Department of Environmental Sciences, Faculty of Natural Sciences, Vytautas Magnus University, Kaunas, Lithuania; 2 Faculty of Medicine, Kauno Kolegija Higher Education Institution, Kaunas, Lithuania

**Keywords:** childbirth experiences, COVID-19 pandemic, childbirth, woman

## Abstract

**INTRODUCTION:**

Childbirth experiences depend on environmental factors, the provision of qualified medical and non-medical care, and the woman's psychological well-being. Stress experienced during pregnancy and childbirth affects a woman's psychological well-being. The aim of this study was to determine the care of women who gave birth during the COVID-19 pandemic in Lithuania.

**METHODS:**

This qualitative study used an interview method to reveal women's childbirth experiences during and before the pandemic in Lithuania. The data obtained during the interview were analyzed using qualitative content analysis. Interviews were taken from 15 women who gave birth at least twice, i.e. the first time until March 2020 (but not earlier than March 2019) and gave birth again during the COVID-19 pandemic (March 2020 – January 2021).

**RESULTS:**

A total of 15 women participanted in the interviews. The experience of childbirth before the COVID-19 pandemic was seen as largely positive by women, but childbirth during the COVID-19 pandemic was mentioned as more complex and negative because of the challenges posed by the pandemic, but easier for other reasons not affected by the pandemic. The results of our study show that a higher proportion of women were satisfied with delivery care in hospitals, were happy, and praised the work of midwives and other staff, which mainly contributed to a positive experience.

**CONCLUSIONS:**

The COVID-19 pandemic posed particular challenges to women's childbirth experiences, and not enough attention was paid to mental health. The stress that was exacerbated during the pandemic period had a profound impact on the pregnant woman in Lithuania.

## INTRODUCTION

Each woman’s birth experience is an individual, complex, emotional event that can drastically change the woman’s life and determine the future of the woman and the child, regardless of whether the experience is more positive or negative^1^. The experience depends on environmental factors, qualified medical and non-medical assistance, and the woman’s psychological well-being. The stress experienced during pregnancy and childbirth affects a woman’s psychological well-being. It has a direct impact not only on the physical health of the mother and the fetus but also on the birth experience of the mother. To improve women’s birth experiences, it is necessary to ensure both smooth medical care and adequate psychological support^2^. According to researchers, the consistency of the relationship and the support received during childbirth is an essential prerequisite for a positive birth experience; the quality of the relationship provides mothers with positive experiences during childbirth. To ensure the mother’s satisfaction, it is necessary to consider the mother’s needs. One of the most frequently expressed needs is emotional support from medical staff and relatives participating in the childbirth^1,2^. The participation and support of relatives is effective childbirth care, which helps the mother to experience more positive emotions, shorten the duration of labor, reduce the need for pain medication and the number of surgical interventions, and avoid neonatal hypoxia. According to the principles of a mother-friendly hospital, all mothers in labor are offered the opportunity to receive continuous emotional and physical support from relatives and qualified woman (dula), and affordable care from professional midwives^3^. The situation and stress caused by the COVID-19 pandemic have changed the quality of maternal and newborn health care worldwide. As a result of this unfavorable situation, women all over the world face limitations that determine the birth experience. Pregnant women and women in labor have to face an unusual situation due to long-term stress, uncertainty, and the emergence of unstable changes in all state institutions. To prevent the spread of the virus, the admission of patients and the flow of visitors are limited in personal health facilities in many countries around the world, the observance of hygiene norms and the use of personal protective equipment are tightened. Such a situation negatively affects the mental health of pregnant women and poses a direct risk to the physical health of the woman and the newborn^4^.

The COVID-19 pandemic was a sensitive issue around the world. To understand and manage this situation, knowing its causes and consequences was essential. The COVID-19 caused by the SARS-CoV-2 virus limited not only the freedom to be with relatives but also the ability to give birth alone in many cases^4^. Researchers are still debating the long-term, potentially transgenerational effects of global, persistent stress on communities, so it is critical to examine its effects on postpartum women as well. New mothers are now forced to cope not only with the already known stress factors of pregnancy, childbirth, and the postpartum period but also to bear the burden of the pandemic, which is even greater if more children are growing in the family^5^.

To understand the problem that arose during COVID-19, so as to create a future evidence base, we aimed to determine the care of women who gave birth during the COVID-19 pandemic in Lithuania.

## METHODS

### Study design and collection of data

This was a qualitative research study. The interview method was chosen to properly and comprehensively analyze and evaluate the participants of the study, women who gave birth before the COVID-19 pandemic and gave birth during it. The data obtained by the interview method are combined and presented in a summarized form.

Interviews were taken from 15 women who gave birth at least twice, i.e. the first time until March 2020 (but not earlier than March 2019) and gave birth again during the COVID-19 pandemic (March 2020 – January 2021). All conversations were recorded on a dictaphone, and the information obtained was systematized, summarized, and transcribed into digital media (Word document) to obtain transparency and clarity of the obtained data.

The interviews were semi-structured, and the main questions were the same for all research participants. However, they were also supplemented with additional, more detailed questions arising during the interviews. In this way, the aim was to obtain as objective and detailed answers as possible and ensure a proper assessment of women’s childbirth experiences. The open-ended interview questions allowed the participants to provide as detailed answers as possible.

The surveys took an average of 30 minutes (the shortest lasted 20 minutes, the longest 50 minutes). The duration depended on the participant’s involvement, eloquence, preparation, and willingness to share information on the questions provided. The time and space for conducting the interviews were coordinated with each participant separately to ensure a suitable, favorable atmosphere and avoid time constraints and other possible distractions. The space for the interview was chosen personally, considering the women’s wishes and ensuring comfort.

The participants who agreed to participate in the research were willing to share information, were sincere, tried to give their answers as clearly as possible, and sought mutual understanding. The study proceeded smoothly due to the benevolence and involvement of the subjects.

The purpose and nature of the research and the use of the interviews, were briefly explained. The introductory part of the questionnaire also contained written information about the research being conducted. It clearly described how to complete the questionnaire and who could participate (selection criteria were indicated). It was emphasized that filling in the questionnaire was not mandatory and could be stopped at any time; anonymity was maintained throughout the research. The aggregated results were used for study purposes only. Subjects were not required to provide their contact details or information that would reveal their identity.

### Participants

The subjects were women who gave birth once or more times before the COVID-19 pandemic and could read and write the Lithuanian language. The sample of subjects was formed by a convenience non-probability sampling method based on the established selection criteria: a woman who has given birth at least twice, both before and during the COVID-19 pandemic.

## RESULTS

The participants were mostly aged 31–40 years. They had higher education, were married, and had two births (Table 1).

**Table 1:** Sociodemographic characteristics of participants who gave birth at least twice between October and November 2022 in Lithuania (N=15)

*Characteristics*	*n*	*%*
**Age** (years)		
≤20	1	6.67
21–30	4	26.67
31–40	10	66.67
**Education level**		
Middle	3	20.00
Higher	4	26.67
Doctoral, etc.	8	53.30
**Marital status**		
Not married	1	6.67
Married	13	87.67
Lives with a partner	1	6.67
**Number of births**		
2	11	73.30
3	3	20.00
≥4	1	6.67

**Figure 1:**
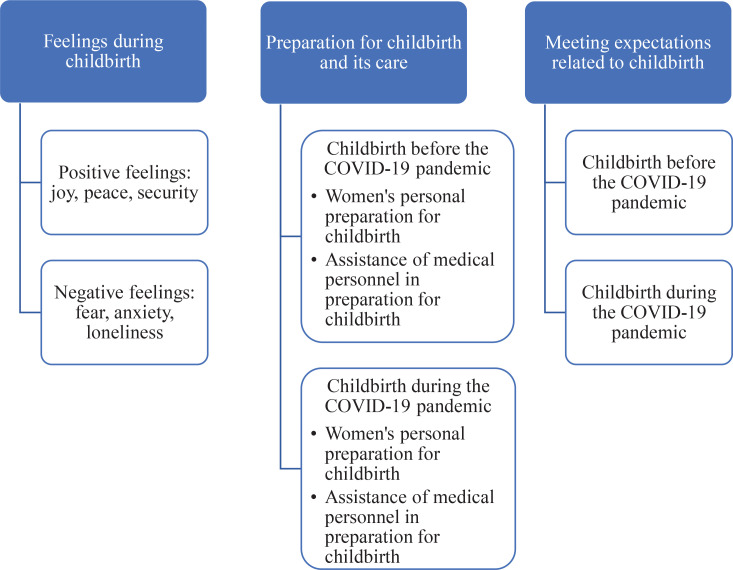
Main themes with sub-themes obtained from the participants at interviews about their experiences of childbirth during the COVID-19 pandemic in Lithuania (N=15)

Analyzing the interviews of the research participants, the main themes (feelings during childbirth; preparation for childbirth and its care; satisfaction of expectations related to childbirth) and sub-themes (positive feelings, negative feelings; women’s preparation for childbirth; the assistance of medical personnel in preparation for childbirth; satisfaction of expectations and failure to meet expectations), were distinguished (Figure 1).

### Feelings during childbirth

Women giving birth for the first time were more often accompanied by fear and anxiety, while women who did not give birth for the first time looked at it with joy, peace, and security. Seven participants chose words such as ‘joy’ and ‘peace’, five participants emphasized the feeling of security, and four participants noted that they felt a sense of fear. Four participants emphasized that they felt anxiety during childbirth. Taking into account the women’s answers, it can be seen that the biggest reasons for anxiety and fear were the first birth:

*‘The birth was the first, unexpected.’*

*‘Because it was the first birth, there was more excitement …’*

lack of information or preparation:

*‘All information, which I knew before, seemed to disappear and everything became very scary.’*

the woman’s psychological characteristics:

*‘I have been a coward all my life, so I remember giving birth with the greatest fear.’*

and the smoothness and mood of the birthing process itself:

*‘Mostly the fear raised the question of whether everything would go smoothly.’*

In the answers of the subjects, such words as ‘I was very afraid’, ‘worried’, ‘there was more excitement’, and ‘we felt very strong anxiety’, stood out. The reasons for positive feelings, based on the women’s answers, were the support and participation of a loved one in the birth, a smooth birth process, the woman’s mood, and not being the first birth. However, positive feelings were more dominant during childbirth before the COVID-19 pandemic, with ten women expressing more positive feelings and five women expressing more negative feelings.

### Preparation for childbirth and its care

The COVID-19 pandemic had complicated the smoothness, quality, and staff services provided in hospitals, thus creating the conditions for the emergence of dissatisfaction with women’s childbirth experiences. Wearing masks, restricting the entry of accompanying persons and taking protective measures, isolating mothers, performing tests, waiting for their results, and the very attitude towards a possibly sick person, were the main factors that led to the emergence of women’s dissatisfaction. Nevertheless, the results of our study show that most women were satisfied with childbirth care in hospitals, were happy, and praised the work of midwives and other staff, which mainly influenced the positive experiences. Although the pandemic raised specific difficulties in medical facilities, women’s satisfaction with childbirth care in hospitals, similar to the period before the pandemic, mainly depended on the staff’s proper care and the warm, pleasant communication with the mothers.

During the interviews, the experiences related to the difficulties encountered during the COVID-19 pandemic came to the fore. The women’s answers showed an increase in negative feelings:

*‘I was very worried whether my husband would be allowed to take part in the birth. I was very afraid of being alone, that no one would help me, I would be left to fate.’*

*‘It was very unpleasant.’*

which were related to the difficulties of testing for the disease:

*‘I had to do prior COVID test. Blood poured from the nose.’*

the fear of contracting COVID-19:

*‘I was extremely worried about the health of myself, my husband and the child.’*

the sense of the unknown, uncertainty about the possibility of the husband participating in the birth:

*‘I was very worried whether he would allow for a man to participate in childbirth.’*

the lack of information:

*‘Is a mask required, a test, is it possible for a man to participate in childbirth, does he need a test.’*

as well as what are the rules after giving birth and remaining in the ward:

*‘Can the man stay, or are several people placed in the ward?’*

and less frequent visits to doctors and organizing part of the work remotely:

*‘Usual but less frequent visits to the attending gynecologist.’*

A lot of attention was paid to the preparation for childbirth in the conditions of enhanced protection due to the pandemic; women had to take additional interest and follow the news regarding the existing rules for the use of personal health protection equipment, the admission of relatives and accompanying persons to the maternity ward:

*‘During this birth, I had to work more independently to find out how things are going births, whether a man can participate, whether masks are mandatory and so on.’*

However, women also shared that the preparation for childbirth was more accessible due to the already existing experience, since childbirth during the COVID-19 pandemic was no longer the first time for all of them:

*‘Since this is my second birth, it was not difficult to prepare, since everything had already passed.’*

*‘Since it was the second birth, I did not take any special steps.’*

and had time to look into aspects related to the difficulties that arose during the first birth and the implementation of changes.

### Meeting expectations related to childbirth

Almost half of the women (8 participants) answered that their expectations were fully met, and they were satisfied with their childbirth experience. The other half of the women (7 participants) answered they had encountered negative experiences. Feelings such as loneliness and sadness prevailed in the women’s answers:

*‘All alone, left to the will of fate. Without any help.’*

*‘I wanted to show my husband the child, but I couldn’t even do that. Which is very, very sad.’*

Considering the women’s answers, it can be concluded that such negative experiences were impressed precisely by the factors caused by the COVID-19 pandemic; the choice of women to give birth with an accompanying person is very limited, most hospitals did not allow relatives or other accompanying persons to the birthing facilities:

*‘I really hoped that a man would participate in the birth and will be with me, he will help me. But I was alone. The midwife kept coming, but she was not a man.’*

*‘I wanted my friend, a doula, to participate, but it didn’t work out.’*

*‘I faced the experience of giving birth alone. This was the most difficult.’*

*‘Only friends rescued with parcels, because the husband was not allowed anywhere.’*

the work and communication of the staff of the medical facility:

*‘The midwife couldn’t even show me how to do a massage on myself. And I constantly felt like a hindrance to the entire staff.’*

*‘She had her own work, that was not with me for a long time. And I really needed someone next to me.’*

*‘I was hoping that the staff would be more understanding, just pay more attention, but I was wrong.’*

and other reasons related to COVID-19.

When meeting the expectations of mothers and fathers, it is essential to distinguish two aspects. Meeting women’s expectations depends on both fixed and variable factors. Our study was also influenced by the fact that women tended to better evaluate childbirth during the COVID-19 pandemic because it was not the first pregnancy; women were more prepared and felt less fear than during the first childbirth. So, these factors were not affected by the pandemic. However, in this case, the changing factors that have affected some women’s birth experiences were restrictions imposed to contain the pandemic, changing medical staff, and other contingencies related to the health of the woman or the child. These changing factors are reflected in women’s responses:

*‘With the experience of giving birth alone. This was the most difficult. I wanted to show my husband the baby, but I couldn’t even do that. Which is very, very sad.’*

## DISCUSSION

The research revealed that women’s feelings and experiences were varied, volatile, changing, and strongly influenced by the number of births.

Women who give birth for the first time mainly experienced negative feelings due to a lack of control, lack of information, and lack of confidence in their own abilities, rather than second-time mothers with previous negative birth experiences. Our study revealed similar data for women giving birth for the first time. The most frequently experienced negative emotions were accompanied by a lack of information and preparation, as well as the woman’s psychological disposition and self-confidence^6^. In contrast to the study of Fenwick^6^, our results on the feelings of non-first-time mothers are mixed, with mostly positive feedback dominating.

Depending on the birth experience, a woman can gain strong self-confidence, or, on the contrary, in the case of a negative birth experience, feelings such as anger, guilt, or even depression can begin to dominate^7^. Therefore, it is very important to monitor the psychological state of the woman, to be able to distinguish the expressed emotions, and to help and support women to contribute to ensuring a positive birth experience.

With the spread of the pandemic, the rapid increase in cases, and the decrease in available hospital beds, hospitals had postponed the provision of non-emergency care in many cases. Most of the assistance provided was for COVID-19 patients, patients requiring intensive care, and women in labor. The women in labor were the main representatives of the healthy human population who were cared for in medical facilities after drastic measures were taken to reduce the spread of the virus, such as limiting the presence of accompanying persons during important life events. Childbirth preparation during the COVID-19 pandemic differed from the pre-pandemic period, not only in terms of more complicated pregnancy care and poorer information dissemination, but also in increased levels of stress for expectant women^5,8^.

Factors such as fear that the mother or newborn might contract COVID-19 during the hospital stay, a reduction or absence of social support and support during the birth and postpartum periods, and a mismatch between pre-pandemic birth expectations and the actual birth experience, could easily had the impact of negative birth experiences and increased feelings of stress during childbirth during the COVID-19 pandemic^5^. A survey of women’s expectations of childbirth during the COVID-19 pandemic revealed more negative feedback than when assessing childbirth before the COVID-19 pandemic.

Ravaldi et al.^8^ suggest that, according to recent studies, women’s expectations and concerns about childbirth have changed significantly due to the COVID-19 pandemic in Italy. Women needed special attention because of the higher amount of stress they experienced. The COVID-19 pandemic has caused much fear, anxiety, and concern among many, not only about the pandemic itself and the disease but also about the limited public health measures to reduce the spread of infection in society. Based on the data of the study conducted by these authors, the following conclusions were drawn regarding women’s expectations and concerns about childbirth at the beginning of the pandemic in Italy: women were equally concerned throughout Italy despite the uneven spread of the COVID-19 cases; women were more likely to worry about other people’s health than their own; women with psychological problems were significantly more concerned about their health because of the pandemic; and the expression of emotions around childbirth changed dramatically before and after the onset of the COVID-19 pandemic^8^.

Therefore, it can be concluded that women whose childbirth experience was not affected by these changing factors tended to evaluate childbirth positively. In contrast, women who faced difficulties during the pandemic evaluated the childbirth experience itself more negatively than positively. However, it cannot be said that in all cases, the negative experience of childbirth was determined by the changes caused by the pandemic because the medical staff also had a significant influence on it.

### Limitations

The study has some limitations. The findings cannot be generalized to the entire country. However, it was difficult to find women who suffered from illnesses during childbirth, and this is why there was no possibility to interview women who suffered from infections during childbirth.

## CONCLUSIONS

When assessing preparation for childbirth during the COVID-19 pandemic, more difficult preparation is seen, complicated by the fear of infection, additional testing for the disease of COVID-19, a sense of uncertainty, lack of information, constant monitoring of changing health protection rules, restriction of the accompanying person’s participation in childbirth, and less frequent visits to those caring for the pregnancy. However, this preparation for childbirth was also easier because childbirth itself was not the first for the women. Therefore, it can be assumed that childbirth during the COVID-19 pandemic was more difficult due to the difficulties caused by the pandemic but easier due to other reasons not affected by it. The situation of the COVID-19 pandemic has complicated the smoothness, quality, and staff services provided in hospitals, thus creating conditions for the emergence of dissatisfaction with women’s childbirth experiences. Wearing masks, restricting the entry of accompanying persons and taking protective measures, isolating mothers, performing tests, waiting for their results, and the very attitude towards a possibly sick person, were the main factors that led to the emergence of women’s dissatisfaction. Nevertheless, the results of our study show that the majority of women were satisfied with childbirth care in hospitals, were happy, and praised the work of midwives and other staff, which mainly influenced the positive experiences.

## Data Availability

The data supporting this research are available from the authors on reasonable request.
